# Deciphering reprogramming efficiency in human induced pluripotent stem cells: insights from the generation of 150 cell lines

**DOI:** 10.3389/fimmu.2025.1719056

**Published:** 2026-01-13

**Authors:** Bernd Kuebler, Silvia Selvitella, Begoña Aran, Angel Raya, Anna Veiga

**Affiliations:** 1 Barcelona Stem Cell Bank, Regenerative Medicine Program, Institut d’Investigació Biomèdica de Bellvitge (IDIBELL), L´Hospitalet de Llobregat, Spain; 2 Program for Clinical Translation of Regenerative Medicine in Catalonia (P-[CMRC]), L´Hospitalet de Llobregat, Spain; 3 Stem Cell Potency Group, Regenerative Medicine Program, Institut d’Investigació Biomèdica de Bellvitge (IDIBELL), L´Hospitalet de Llobregat, Spain; 4 Center for Networked Biomedical Research on Bioengineering, Biomaterials and Nanomedicine (CIBER-BBN), Madrid, Spain; 5 Physiological Sciences Department, Faculty of Medicine and Health Sciences, University of Barcelona, L´Hospitalet de Llobregat, Spain; 6 Institució Catalana de Recerca i Estudis Avançats (ICREA), Barcelona, Spain

**Keywords:** induced pluripotent stem cells, integrating reprogramming methods, iPSC characterization, non-integrating reprogramming methods, reprogramming efficiency

## Abstract

**Introduction:**

The discovery of induced pluripotent stem cells (iPSCs) revolutionized the field of translational medicine by enabling the reprogramming of adult somatic cells into a pluripotent state. From personalized disease models to innovative cell therapies, iPSCs are poised to play a central role in the future of clinical medicine. iPSCs hold enormous promises due to their ability to self-renew indefinitely and differentiate into all somatic cell types, thus offering patient-specific cellular models and therapeutic options without the ethical constraints of embryonic stem cells (ESCs). iPSCs, which exhibit pluripotency similar to embryonic stem cells, are generated by introducing specific factors into terminally differentiated cells, inducing a shift in their epigenetic and transcriptional landscape, which leads to the reactivation of the pluripotency program of the cells. Nevertheless, the mechanisms underlying successful reprogramming remain poorly understood.

**Methods:**

In this study we performed a statistical evaluation of reprogramming efficiencies of 150 iPSC lines generated in our lab, comparing factors such as the starting somatic cell type, passage number, donor´s health status, donor age and sex, reprogramming methodology, and growth conditions.

**Results/Discussion:**

We found that the most relevant factor influencing reprogramming efficiency is the developmental status of the starting cells. While other parameters may exert minor effects, inherent donor-specific biological characteristics appear to play the strongest role in determining reprogramming outcomes.

## 1 Introduction

The concept of cellular reprogramming emerged from pioneering work by Kazutoshi Takahashi and Shinya Yamanaka in 2006, when they identified four transcription factors—Oct4, Sox2, Klf4, and c-Myc (OSKM)- as key regulators of pluripotency. These factors were introduced into mouse fibroblasts, where they induced a reversion to a pluripotent state, resulting in the generation of iPSCs (1). One year later iPSCs were generated from human fibroblasts by retroviral transduction with OSKM (2) and by lentiviral transduction with OCT4, SOX2, NANOG, and LIN28 (3). Lentiviral or retroviral delivery of the reprogramming factors leads to their integration into the genome and thus stable expression for iPSC induction (4).

Since then, many techniques have been published about how to deliver the transcription factors into cells (5, 6), including generation of iPSC using transposons (7, 8), bacteriophages (9) or Zink finger nucleases (10).

These reprogramming methods have raised major concerns for their use in therapy, primarily due to the risk of insertional mutagenesis and the potential for the incomplete silencing or residual expression of reprogramming factors caused by the integration of DNA into the host genome.

The genomic instability resulting from integrating methodologies led to the development of non-integrating techniques to optimize the efficiency, safety, and versatility of reprogramming.

Non-Integrating iPSC have been generated using adenovirus (11, 12), Sendai virus (13–16), episomal plasmids (17, 18), RNA (19–22), synthetic self-replicative RNA (23), miRNA (24–26), minicircle DNA (18, 27) and by CRISPR activation (28, 29).

Alternative ways to generate iPSCs consist in the direct delivery of recombinant proteins into the somatic cells (30, 31) or reprogramming by adding small molecules and chemical compounds to the cell culture medium, to circumvent exogenously expressed transcription factors (32–35).

Reprogramming somatic cells to a pluripotent state involves resetting the cellular epigenome and transcriptional profile to resemble that of pluripotent cells by reactivating genes associated with pluripotency and the silencing of genes that maintain somatic cell identity (36). The key transcription factors OSKM are primarily responsible for initiating these changes by binding to chromatin remodeling complexes to reconfigure the epigenome, a critical step in resetting cellular identity. This interaction leads to the induction of a cascade of molecular events, including DNA demethylation, histone modification, and chromatin remodeling (4). The cellular metabolism is also altered during reprogramming, shifting from oxidative phosphorylation to glycolysis, like ESCs (37).

In an immunological and alloimmune context, reprogramming efficiency can be relevant because it reflects donor-specific immune and inflammatory states that can influence cellular plasticity and iPSC generation. Variability in efficiency may therefore indicate underlying immunological differences that affect downstream differentiation and the interpretation of alloimmune responses. This concept aligns with studies showing that innate immune activation and inflammatory signaling modulate somatic cell reprogramming efficiency (38).

The Barcelona Stem Cell Bank (Banco de Lineas Celulares de Barcelona, BLC-B) is a functional unit of the Regenerative Medicine Program at the Institut d’Investigació Biomèdica de Bellvitge (IDIBELL), offering services for the generation, characterization, and banking of human iPSC and human ESCs. Over 150 lines starting from different human somatic cell types, such as skin derived fibroblasts and keratinocytes, peripheral blood mononuclear cells (PBMCs), CD34 positive cord blood cells, tumor cells, and urine cells were successfully reprogrammed to iPSCs. The somatic cells were obtained from healthy donors as well as from over 100 donors suffering a broad range of diseases. The iPSCs were generated using integrating (retrovirus and lentivirus-based) and non-integrating (Cytotune™-iPS 2.0 Sendai Reprogramming Kit (SeV 2.0), CTS Cytotune™-iPS 2.1 Sendai Reprogramming Kit (SeV 2.1), episomal plasmids (ep), and mRNA-based) techniques. However, the process of reprogramming remains inherently inefficient and can be influenced by different biological and technical variables.

In this study a comprehensive statistical evaluation of reprogramming efficiencies using one of the largest datasets to date-the second-highest number of samples analyzed in this context. By simultaneously assessing factors such as as starting somatic cell type, reprogramming methodology, passage number, donor health status, donor age and sex, and growth conditions across a diverse donor pool, we evaluated these variables within a unified framework. This approach provides insights into the key determinants that influence successful iPSC generation.

Understanding and quantifying the reprogramming efficiency is essential to optimize protocols, improve yield, and ensure the quality and genomic integrity of the resulting iPSC lines. Moreover, identifying key variables that impact the success of iPSC derivation is essential to understand the reprogramming process and to refine strategies that enhance both efficiency and reproducibility.

## 2 Materials and methods

### 2.1 Materials

A total of 150 iPSC lines were generated using various reprogramming technologies, including both integrating and non-integrating methods. In general, human fibroblasts were reprogrammed by retroviral delivery of Yamanaka factors, by nucleofection with episomal plasmids, by Sendai virus transduction or by transfection with non-modified RNAs. Fibroblasts were transduced, nucleofected or transfected at different passages between 3 and 12, with passages <5 considered early and passages >5 considered late. Peripheral blood mononuclear cells (PBMCs) and CD34 positive cells isolated from human cord blood units (CD34+) were reprogrammed by Sendai virus transduction.

The most commonly used methodology was Sendai virus, accounting for 56.7% of the lines (including SeV 2.0 and SeV 2.1). This was followed by episomal plasmids, used in 33.3% of the lines. The remaining lines were generated using mRNA (3.33%) and retroviral vectors (7.3%) (**Table 1**).

**Table 1:** Summary table with variable data used in the analysis of reprogramming efficiency.

Characteristic	N = 150^1^
Sex	
Female	63 (42%)
Male	87 (58%)
Status	
Disease	116 (77%)
Healthy	34 (23%)
Cell type	
CB/CD34+	10 (6.7%)
EPCs	1 (0.7%)
EUC	1 (0.7%)
Fibroblasts	78 (52%)
PBMCs	57 (38%)
Schwannoma	3 (2.0%)
Reprogramming methodology	
Episomal plasmids	49 (33%)
mRNA	5 (3.3%)
Retro virus	11 (7.3%)
Sendai virus 2.0	75 (50%)
Sendai virus 2.1	10 (6.7%)
Age average: 35.89 (SD: 24.59), [min. age: 0.00, max. age: 107.00]	
Age groups:	
Young (<30)	55 (39%)
Adult (30-60)	65 (46%)
Elderly (>60)	20 (14%)
^1^n (%)	

Values are presented as n (%) for categorical variables and as mean (SD) for continuous variables.

Several human cell types were used as starting material. Fibroblasts (52%) and peripheral blood mononuclear cells (PBMCs, 38%) were the most frequently used. The third most represented cell type in our collection was cord blood-derived CD34+ cells, which were specifically used to generate a subset of cell therapy-intended lines, selected to represent the most common haplotypes in the Spanish population (39). Additionally, we reprogrammed endothelial progenitor cells (EPCs) (22), epithelial urine cells (EUCs), and schwannoma cells.

Among the 150 iPSC lines, 42% were derived from female donors and 58% from male donors. Regarding donor age, the lines were generated from individuals ranging from newborns to 107 years old. Age distribution was well represented across groups: 39% of the lines were derived from young individuals (0–30 years), 46% from adults (30–60 years), and 14% from elderly individuals (>60 years) (**Table 1**).

Given that one of the main objectives of iPSC technology is disease modeling, 116 lines (77%) were derived from patients diagnosed with 33 different diseases. The remaining 34 lines (23%) were obtained from healthy individuals and are typically used as controls in disease modeling assays (**Table 1**).

### 2.2 Reprogramming methods

As the BLC-B is a platform providing services for the generation, characterization, and banking of human iPSCs and ESCs, the selection of somatic cell types for reprogramming, as well as the reprogramming methodologies employed, was determined by the principal investigators leading each individual project.

#### 2.2.1 Integrating reprogramming by retroviral transduction

Skin derived human fibroblasts were reprogrammed using two polycistronic retroviruses encoding the four Yamanaka factors OCT4, SOX2, KLF4, and c-MYC. Retroviruses were produced in Phoenix Amphotrophic cells following transfection with pMX-OCT4_Flag-VP16-PTV-Sox2_HA-Orange or pMX-KLF4-cMYC-GFP vectors. Retrovirus containing medium from both approaches was collected 48 h post-transfection and used to transduce 1x10^5^ cells via spin infection (40, 41). After 3 days, transduced fibroblasts were trypsinized and seeded onto irradiated human foreskin fibroblasts in hES medium (Knockout DMEM with 20% Knockout serum replacement, 2 mM Glutamax, 1% penicillin–streptomycin, 0.1 mM β-mercaptoethanol, 1% non-essential amino acids (NEAA) (all Gibco), and 10 ng/ml bFGF (Millipore)) and incubated in a humified incubator at 37°C and 5% CO_2_. Transduction efficiency was assessed by monitoring GFP- and mOrange-positive cells.

After emerging of iPSC colonies around day 14 after transduction, single iPSC clones were manually picked for expansion. From passage 5 on, clones were changed to feeder-free conditions on plates previously coated with 23ug/cm^2^ matrigel (Corning) and mTSeR1 medium (StemCell Technologies).

#### 2.2.2 Non-integrating reprogramming methods

##### 2.2.2.1 Episomal reprogramming

For non-integrating EPISOMAL reprogramming of human fibroblasts, 3 OriP/EBNA1 episomal vectors expressing OCT3/4, SOX2, KLF4, Lin28, L-Myc and a p53 knock down shRNA were used (Addgene, #27080, #27078, #27077). 5.0x 10^5^ cells in 100ul nucleofection solution (Lonza, Amaxa NHDF Nucleofector Kit, #VPD-1001) were nucleofected with the Amaxa Nucleofector II device (Lonza) applying the U-023 program (42, 43). Cells were seeded in matrigel coated wells of a 6well plate in DMEM medium supplemented with 10% Hyclone FBS, 2 mM Glutamax, and 1% penicillin–streptomycin (DMEM complete).

In parallel, 5.0x 10^5^ cells were nucleofected with an episomal plasmid carrying eGFP (Addgene, #27082) as described above. Nucleofection efficiency was determined 72 h after nucleofection by measuring the percentage of GFP-positive cells using flow cytometry (FACS).

At day 7 after nucleofection, fibroblasts were trypsinized and seeded onto matrigel coated 6 well plates in ReproTeSR medium (Stemcell Technologies). From residual nucleofected cells, cell pellets were prepared, and genomic DNA (gDNA) as well as RNA were extracted and used as positive controls for subsequent molecular characterization. The wells containing the nucleofected cells were monitored daily using a stereomicroscope to identify the emergence of cell clusters adherent to the culture surface, indicative of successful reprogramming.

On average, first iPSC colonies appeared 16 days after nucleofection of the fibroblasts. Clones were manually picked, changed to mTeSR1 medium (Stemcell Technologies) and passaged for expansion, cryopreservation and characterization.

##### 2.2.2.2 SeV reprogramming

Human fibroblasts were reprogrammed using the CytoTune™-iPS 2.0 Sendai Reprogramming Kit (Thermo Fischer Scientific). 1x 10^5^ cells were seeded in 6 wells of a 6 well plate in DMEM complete. The next day, cells from 1 well were trypsinized and counted to determine the number of cells in the other wells. Between 3 × 10^5^ and 5 × 10^5^ cells were transduced with the three virus constructs included in the kit—encoding KOS, c-MYC, and KLF4—at multiplicities of infection (MOI) of 5:5:3 (KOS:c-MYC: KLF4) in DMEM complete supplemented with 4 μg/ml polybrene. After 24 hours, the medium was replaced with fresh DMEM complete. At day 7 after transduction, transduced fibroblasts were trypsinized and counted. Cells were seeded on wells of 6 well plates previously coated with matrigel in ReproTeSR medium. Cell pellets were prepared from remaining transduced cells, and RNA was extracted for use as a positive control in molecular characterization PCR.

On average, first iPSC colonies emerged 12 days after transduction of fibroblasts. Individual clones were manually picked, switched to mTeSR1 medium and passaged for expansion, cryopreservation, and characterization.

PBMCs were reprogrammed using the CytoTune™-iPS 2.0 Sendai Reprogramming Kit (Thermo Fischer Scientific). A total of 5 × 10^5^ cells were transduced with the three virus constructs included in the kit—encoding KOS, c-MYC, and KLF4—at a MOI of 5:5:3 (KOS:c-MYC: KLF4). Transductions were carried out in 1.5 ml of StemPro 34 Serum Free Medium supplemented with 1X Glutamax (SP34 complete medium), cytokines (100 ng/ml Stem Cell Factor, SCF; 100 ng/ml Fms-related tyrosine kinase 3 ligand, Ftl3L; 20 ng/ml Interleukin-3, IL-3; and 20 ng/ml Interleukin-6, IL-6, all Peprotech) and 4 μg/ml polybrene. Twenty-four hours after transduction, cells were seeded in SP34 complete medium supplemented with cytokines in an ultra-low attachment plate at 37°C and 5% CO_2_. After 3 days cells were changed to matrigel coated 6 well plates in SP34 complete medium for another 3 days. In parallel, a cell pellet was prepared, from which RNA was extracted and used as a positive control for molecular characterization. 7 days after transduction the medium was changed to ReproTeSR medium (41). On average, first iPSC colonies emerged 9 days after transduction of PBMCs. Clones were manually picked, switched to mTeSR1 medium and passaged for expansion, cryopreservation, and characterization.

CD34-positive cells isolated from human cord blood units were reprogrammed using the CTS CytoTune™-iPS 2.1 Sendai Reprogramming Kit (Thermo Fisher Scientific) (39). Briefly, 1 × 10^4^ CD34 + cells were transduced with the three viral constructs provided in the kit—encoding KOS, L-MYC, and KLF4—at a MOI of 5:2.5:3 (KOS:L-MYC: KLF4) in SP34 complete medium supplemented with cytokines (50 ng/ml SCF; 50 ng/ml FLT3L; 10 ng/ml thrombopoietin; and 10 ng/ml IL-6), and 4 μg/ml polybrene. The transductions were performed overnight at 37°C in a humidified atmosphere containing 5% CO_2_. The next day, infected CD34 + cells were seeded on laminin 521 (Biolamina, Art. No. CT521) coated dishes in SP34 complete medium supplemented with cytokines. After 3 days, the medium with floating cells was changed to SP34 complete medium without cytokines by spinning. From day 6 after infection, the medium was changed to Essential 8 Flex Medium (Thermo Fisher, Art. No. A2858501), and the wells were observed every day under a stereomicroscope for the emergence of clusters of attached cells indicative of reprogrammed cells.

First colonies emerged around day 8. Approximately 16 to 18 days after transduction, single colonies were manually picked and seeded in Essential 8 Flex medium on laminin coated dishes. From residual Sendai virus-infected cells, cell pellets were prepared, RNA was extracted and used as a positive control in the PCR performed to prove the absence of Sendai virus in the generated iPSC clones.

##### 2.2.2.3 RNA reprogramming

Skin derived human fibroblasts were reprogrammed using the StemRNA 3^rd^ Generation Reprogramming Kit (Stemgent). 7,5x 10^4^ cells were seeded in DMEM complete medium on matrigel coated 6 well plates. The day after, cells were transfected with an RNA mixture of synthetic, non-modified reprogramming factors (OCT4, SOX2, KLF4, cMYC, NANOG, and LIN28), immune evasion mRNA (E3, K3, B18), and mature, double-stranded microRNA clusters from the 302/367 cluster in ReproTeSR medium at 37°C, 5% CO_2_ and 5% O_2_. A total of 4 transfections were performed on 4 consecutive days and cells were cultured under hypoxic conditions.

First colonies emerged around day 10 after the first transfection. 10 single clones were manually picked and seeded in mTeSR1 medium on matrigel coated dishes and passaged for expansion, cryopreservation, and characterization.

### 2.3 Experimental timeline and characterization of generated iPSCs

The overall workflow for the generation, characterization and banking of iPSCs is described below: Following the emergence of initial colonies after nucleofection, transduction, or transfection, 10 to 14 single clones were manually picked and expanded. A mycoplasma test was performed from all clones and cells were frozen at low passages (between p3 and p4). 4 to 6 clones were selected according to morphological criteria, like compact colonies with smooth, well-defined borders, tightly packed cells with a large nucleus and scant cytoplasm and further expanded. At passages around p7, cell pellets were prepared and gDNA and/or RNA were extracted to perform the molecular characterization. For clones derived by non-integrating reprogramming approaches, karyotypic analyses were conducted on two transgene-free clones, from which one was selected for comprehensive downstream characterization. The full characterization of hiPSCs was performed using the methodology previously described (44), with some modifications, based on the ISSCR guidelines (45). The characterization includes immunocytochemistry (ICC) for undifferentiated state markers, alkaline phosphatase (AP) staining, differentiation *in vitro* by embryoid body (EB) formation and ICC and determination of short tandem repeats (STR). A second mycoplasma test was performed before banking. Karyotype determination was repeated after banking.

### 2.4 Banking of generated clones. DNA fingerprinting and karyotype analysis.

For banking, iPSCs were cryopreserved when cultures reached approximately 70–90% confluency and displayed minimal spontaneous differentiation, at a cell density of around 2× 10^6^ cells/tube. Prior to banking, cell lines were confirmed to be mycoplasma-free, a G-banding karyotype, and STR analysis were performed. After banking, nearly all of the iPSC lines used in this study have been registered with the Spanish Health Institute Carlos III (ISCIII), https://www.isciii.es/en/servicios/biobancos/banco-nacional-lineas-celulares/lineas-ips and with the Human Pluripotent Stem Cell Registry https://hpscreg.eu/. The remaining lines are currently in the process of being registered.

### 2.5 Statistical Analysis.

Reprogramming efficiencies were calculated using the following formula:


$$Reprogramming\;Efficiency=\frac{\#\;Emerging\;colonies}{\#\;somatic\;cell\;seeded\;}\;\times 100$$

Descriptive statistics, including mean, standard deviation (SD), and median values, were calculated for reprogramming efficiencies across all groups to summarize the data distribution and support subsequent statistical analyses (**Table 2**).

**Table 2:** Summary of reprogramming efficiency (%) according to different biological and methodological variables.

Variable		Reprogramming efficiency (%)		
		Mean	Standart deviation (SD)	Median
Cell Type	CB/CD34+	1.199	0.529	1.180
	Fibroblasts	0.040	0.103	0.007
	PBMCs	0.015	0.013	0.009
	Schwannoma	0.121	0.158	0.060
Sex	Female	0.029	0.091	0.009
	Male	0.033	0.075	0.007
Age	Young (<30 years)	0.039	0.088	0.009
	Adult (30–60 years)	0.027	0.088	0.006
	Elderly (>60 years)	0.024	0.019	0.019
Status	Healthy	0.066	0.116	0.029
	Disease	0.024	0.071	0.008
Methodology (All cell types)	Episomal Plasmids	0.017	0.027	0.006
	mRNA	0.177	0.226	0.052
	Retrovirus	0.030	0.029	0.020
	Sendai Virus	0.031	0.087	0.010
Methodology(Only Fibroblast)	Episomal Plasmids	0.017	0.027	0.006
	mRNA	0.221	0.235	0.191
	Retrovirus	0.030	0.030	0.020
	Sendai Virus	0.076	0.176	0.031
Passage	Early	0.032	0.110	0.007
	Late	0.049	0.096	0.010

Values are presented as mean, standard deviation (SD), and median. Efficiency was calculated for each group independently. “Methodology (Only Fibroblast)” refers to reprogramming methods applied exclusively to fibroblast-derived cells. “Passage” indicates the stage of cell culture at the time of reprogramming (early (<5) vs. late (>5).

Before performing statistical analyses, reprogramming efficiencies were normalized to improve the distributional properties of the data, reduce the influence of outlier values, and minimize overall variability. The transformation applied was:


Normalized Efficiency=LN((Reprogramming Effiency×1000)+1)

Statistical analyses were conducted using R, version 4.3.1. The predictor variables analyzed included: starting cell type, reprogramming methodology (both across all cell types and specifically within fibroblasts), health status (Healthy vs Disease), gender, age (Young <30 years, Adult 30–60 years, Elderly >60 years), and passage number. The response variable used was Normalized Efficiency.

Prior to applying statistical tests, the assumptions of ANOVA were evaluated. Normality was assessed using the Shapiro-Wilk test. For variables that did not meet the normality assumption, a non-parametric Kruskal-Wallis test was applied. For variables with normal distribution, Levene’s test was used to assess homogeneity of variances. Variables that satisfied both assumptions were analyzed using ANOVA. In cases where normality was present but homogeneity of variance was violated, Welch’s ANOVA was used instead.

For variables showing significant differences (p < 0.05) in ANOVA, Welch’s ANOVA, or Kruskal-Wallis tests, *post-hoc* analyses were performed to identify specific group differences. For *post-hoc* analyses, all tests included adjustments for multiple comparisons to control the family-wise error rate. Specifically, Tukey’s test (following ANOVA) and Games-Howell (following Welch’s ANOVA) apply built-in corrections for pairwise comparisons. For Dunn’s test (following Kruskal-Wallis) a Holm correction was applied.

Statistical significance was annotated using the following codes:

**** for p < 0.0001, *** for p < 0.001, ** for p < 0.01, and * for p < 0.05.

To minimize confounding between cell type and reprogramming method, CD34+ samples were used only in the comparison of the starting cell type. CD34+ cells were exclusively reprogrammed using the CTS Cytotune™-iPS 2.1 Sendai reprogramming Kit and exhibit markedly higher efficiencies. Therefore CD34+ cells were excluded from the other comparisons to avoid bias. Analyses were then performed on fibroblasts and PBMCs, which allowed comparisons across methods and donor-related variables.

To further analyze and understand the potential confounding effects of certain variables, complementary analysis was performed using Generalized Linear Models (GLMs). Based on the distribution of the response variable: efficiency (**Supplementary Figure 1A**) and the mean–variance relationship (**Supplementary Figure 1B**), the model was fitted using a Gamma distribution with a log link. Additionally, three separate analyses were conducted to better explore the dataset. First, the full dataset was used to evaluate the effect of CB CD34+ cells (**Tables 3**, **4**) and the model was adjusted to a quasi-Gamma distribution due to overdispersion (**Supplementary Figure 2**). Second, to avoid potential bias introduced by CB CD34+ cells, which showed markedly higher reprogramming efficiencies, a model excluding these samples was performed (**Tables 5**–**8**). Finally, only the efficiencies of lines generated from fibroblasts were analyzed (**Tables 9**–**11**), as this cell type was reprogrammed using multiple methodologies and across different passages, allowing for a more balanced comparison.

**Table 3:** Results of GLM fitted quasi-Gamma distribution and log link using all data, including CB CD34 +.

Predictor	Estimate	Std. Error	t-value	p-value	Significance
(Intercept)	-2.2589	0.637	-3.546	0.000535	n.s
Sex: Male	-0.1537	0.2162	-0.711	0.478333	n.s
Age: Elderly	0.3447	0.3388	1.017	0.310733	n.s
Age: Young	-0.205	0.245	-0.837	0.404164	n.s
Cell type: EPCs	-7.6203	1.5211	-5.01	1.65E-06	***
Cell type: EUCs	-2.804	1.3988	-2.005	0.046987	*
Cell type: Fibroblasts	-2.0165	0.574	-3.513	0.0006	***
Cell type: PBMCs	-3.7613	0.5459	-6.89	1.86E-10	***
Cell type: Schwannoma	-1.2269	0.8739	-1.404	0.162588	n.s
Methodology: mRNA	2.1185	0.6991	3.03	0.002923	**
Methodology: Retro	0.5496	0.4288	1.282	0.202086	n.s
Methodology: SeV	1.7326	0.3647	4.751	5.06E-06	***
Health Status: Healthy	1.0067	0.3195	3.151	0.002	**

Intercept represents the estimated efficiency for the reference levels: CB CD34+ (Cell type), Episomal Plasmids (Methodology), Female (Sex), Disease donors (Health status) and Adult (Age). Estimates for other levels are expressed as differences relative to this baseline. Signif. codes: <0.001 ‘***’ <0.01 ‘**’ <0.05 ‘*’. Estimates are on the log scale; exponentiate for interpretation on the original scale.

**Table 4:** Pairwise comparisons of estimated marginal means obtained from GLM models of cell type including CB CD34+ data.

Contrast	Estimate	SE	df	Lower CI (95%)	Upper CI (95%)	z- or t- ratio	p-value	Significance
CB CD34+ - EPCs	7.62	1.52	Inf	3.29	11.955	5.01	0.0001	***
CB CD34+ - EUC	2.804	1.4	Inf	-1.18	6.79	2.005	0.3396	n.s
CB CD34+ - Fibroblasts	2.016	0.574	Inf	0.39	3.652	3.513	0.0059	**
CB CD34+ - PBMCs	3.761	0.546	Inf	2.21	5.317	6.89	0.0001	***
CB CD34+ - Schwannoma	1.227	0.874	Inf	-1.26	3.717	1.404	0.7247	n.s
EPCs - EUC	-4.816	1.9	Inf	-10.23	0.603	-2.533	0.1146	n.s
EPCs - Fibroblasts	-5.604	1.39	Inf	-9.56	-1.652	-4.041	0.0008	***
EPCs - PBMCs	-3.859	1.43	Inf	-7.92	0.203	-2.707	0.0737	n.S
EPCs – Schwannoma	-6.393	1.6	Inf	-10.96	-1.821	-3.985	0.001	**
EUC - Fibroblasts	-0.787	1.32	Inf	-4.56	2.981	-0.595	0.9914	n.s
EUC – PBMCs	0.957	1.37	Inf	-2.95	4.864	0.698	0.9822	n.s
EUC - Schwannoma	-1.577	1.56	Inf	-6.03	2.88	-1.008	0.9154	n.s
Fibroblasts – PBMCs	1.745	0.365	Inf	0.7	2.785	4.782	0.0001	***
Fibroblasts - Schwannoma	-0.79	0.783	Inf	-3.021	1.442	-1.008	0.9154	n.s
PBMCs - Schwannoma	-2.534	0.749	Inf	-4.67	-0.4	-3.383	0.0094	**

Each row represents the contrast between two levels of the factor of interest. ‘Estimate’ indicates the difference in adjusted means (on the log scale), with its standard error (SE). 95% confidence intervals (CI) and p-values are provided. P-values were adjusted for multiple comparisons using Tukey’s procedure. Positive estimates indicate higher efficiency in the first group compared to the second. For interpretation on the original scale, exponentiate the estimates. Signif. codes: <0.001 ‘***’ <0.01 ‘**’ <0.05 ‘*’

**Table 5:** Results of the GLM fitted Gamma distribution and log link using the data without the CB CD34+ efficiencies.

Predictor	Estimate	Std. Error	t-value	p-value	Significance
(Intercept)	-4.2711	0.2356	-18.13	2E-16	–
Sex: Male	-0.1656	0.2315	-0.715	0.475768	n.s
Age: Elderly	0.3471	0.349	0.995	0.321812	n.s
Age: Young	-0.2004	0.2527	-0.793	0.429353	n.s
Cell type: EPCs	-5.5991	1.4286	-3.919	0.000144	***
Cell type: EUCs	-0.7943	1.3627	-0.583	0.561006	n.s
Cell type: PBMCs	-1.7416	0.3758	-4.634	8.7E-06	***
Cell type: Schwannoma	0.7956	0.8066	0.986	0.325856	n.s
Methodology: mRNA	2.116	0.7201	2.938	0.003916	**
Methodology: Retro	0.5467	0.4416	1.238	0.218008	n.s
Methodology: SV	1.7295	0.3756	4.604	9.84E-06	***
Health Status: Healthy	1.0121	0.3296	3.071	0.002606	**

Intercept represents the estimated efficiency for the reference levels: Fibroblasts (Cell type), Episomal Plasmids (Methodology), Female (Sex), Disease donors (Health status) and Adult (Age). Estimates for other levels are expressed as differences relative to this baseline. Signif. codes: <0.001 ‘***’ <0.01 ‘**’ <0.05 ‘*’. Estimates are on the log scale; exponentiate for interpretation on the original scale.

**Table 6:** Pairwise comparisons of estimated marginal means obtained from GLM models of Cell type without CB CD34+ data.

Contrast	Estimate	SE	df	Lower CI (95%)	Upper CI (95%)	t-ratio	p-value	Significance
Fibroblasts - EPCs	5.599	1.43	128	1.646	9.552	3.919	0.0013	**
Fibroblasts - EUC	0.794	1.36	128	-2.976	4.565	0.583	0.9774	n.s
Fibroblasts – PBMCs	1.742	0.376	128	0.702	2.781	4.634	0.0001	***
Fibroblasts - Schwannoma	-0.796	0.807	128	-3.027	1.436	-0.986	0.8611	n.s
EPCs – EUC	-4.805	1.96	128	-10.228	0.618	-2.452	0.1085	n.s
EPCs - PBMCs	-3.858	1.47	128	-7.92	0.205	-2.627	0.0714	n.s
EPCs – Schwannoma	-6.395	1.65	128	-10.967	-1.823	-3.87	0.0016	**
EUC - PBMCs	0.947	1.41	128	-2.961	4.856	0.671	0.9624	n.s
EUC – Schwannoma	-1.59	1.61	128	-6.05	2.871	-0.986	0.8611	n.s
PBMCs - Schwannoma	-2.537	0.772	128	-4.673	-0.402	-3.288	0.0112	*

Each row represents the contrast between two levels of the factor of interest. ‘Estimate’ indicates the difference in adjusted means (on the log scale), with its standard error (SE). 95% confidence intervals (CI) and p-values are provided. P-values were adjusted for multiple comparisons using Tukey’s procedure. Positive estimates indicate higher efficiency in the first group compared to the second. For interpretation on the original scale, exponentiate the estimates. Signif. codes: <0.001 ‘***’ <0.01 ‘**’ <0.05 ‘*’

**Table 7:** Pairwise comparisons of estimated marginal means obtained from GLM models of methodology without CB CD34+ data.

Contrast	Estimate	SE	df	Lower CI (95%)	Upper CI (95%)	t-ratio	p-value	Significance
Episomal – mRNA	-2.116	0.72	128	-3.991	-0.241	-2.938	0.0202	*
Episomal – Retro	-0.547	0.442	128	-1.696	0.603	-1.238	0.604	n.s
Episomal – SV	-1.73	0.376	128	-2.707	-0.752	-4.604	0.0001	***
mRNA - Retro	1.569	0.789	128	-0.484	3.623	1.989	0.1974	n.s
mRNA – SV	0.386	0.762	128	-1.597	2.37	0.507	0.9573	n.s
Retro - SV	-1.183	0.516	128	-2.525	0.16	-2.294	0.1049	n.s

Each row represents the contrast between two levels of the factor of interest. ‘Estimate’ indicates the difference in adjusted means (on the log scale), with its standard error (SE). 95% confidence intervals (CI) and p-values are provided. P-values were adjusted for multiple comparisons using Tukey’s procedure. Positive estimates indicate higher efficiency in the first group compared to the second. For interpretation on the original scale, exponentiate the estimates. Signif. codes: <0.001 ‘***’ <0.01 ‘**’ <0.05 ‘*’

**Table 8:** Pairwise comparisons of estimated marginal means obtained from GLM models of Age without CB CD34+ data.

Contrast	Estimate	SE	df	Lower CI (95%)	Upper CI (95%)	t-ratio	p-value	Significance
Adult - Elderly	-0.347	0.349	128	-1.175	0.48	-0.995	0.5816	n.s
Adult - Young	0.2	0.253	128	-0.399	0.8	0.793	0.7081	n.s
Elderly - Young	0.547	0.378	128	-0.349	1.44	1.448	0.3194	n.s

Each row represents the contrast between two levels of the factor of interest. ‘Estimate’ indicates the difference in adjusted means (on the log scale), with its standard error (SE). 95% confidence intervals (CI) and p-values are provided. P-values were adjusted for multiple comparisons using Tukey’s procedure. Positive estimates indicate higher efficiency in the first group compared to the second. For interpretation on the original scale, exponentiate the estimates. Signif. codes: <0.001 ‘***’ <0.01 ‘**’ <0.05 ‘*’

**Table 9:** Results of the GLM fitted Gamma distribution and log link using only the data of the cell type fibroblasts.

Predictor	Estimate	Std. Error	t-value	p-value	Significance
(Intercept)	-4.3212	0.3072	-14.064	2E-16	–
Passage: Late	0.3516	0.4231	0.831	0.40872	n.s
Methodology: mRNA	2.4611	0.9093	2.707	0.00846	**
Methodology: Retro	0.7959	0.6048	1.316	0.19234	n.s
Methodology: SV	1.6284	0.5131	3.174	0.0022	**

Intercept represents the estimated efficiency for the reference levels: Early (Passage) and Episomal Plasmids (Methodology). Estimates for other levels are expressed as differences relative to this baseline. Signif. codes: <0.001 ‘***’ <0.01 ‘**’ <0.05 ‘*’. Estimates are on the log scale; exponentiate for interpretation on the original scale.

**Table 10:** Pairwise comparisons of estimated marginal means obtained from GLM models of Methodology using only fibroblasts data.

Contrast	Estimate	SE	df	Lower CI (95%)	Upper CI (95%)	t-ratio	p-value	Significance
Episomal - mRNA	-2.461	0.909	73	-4.85	-0.0703	-2.707	0.0412	*
Episomal - Retro	-0.796	0.605	73	-2.39	0.7943	-1.316	0.5559	n.s
Episomal – SV	-1.628	0.513	73	-2.98	-0.2794	-3.174	0.0116	*
mRNA - Retro	1.665	1.07	73	-1.15	4.4786	1.556	0.4098	n.s
mRNA – SV	0.833	0.958	73	-1.69	3.3511	0.869	0.8206	n.s
Retro - SV	-0.833	0.74	73	-2.78	1.1138	-1.125	0.6756	n.s

Each row represents the contrast between two levels of the factor of interest. ‘Estimate’ indicates the difference in adjusted means (on the log scale), with its standard error (SE). 95% confidence intervals (CI) and p-values are provided. P-values were adjusted for multiple comparisons using Tukey’s procedure. Positive estimates indicate higher efficiency in the first group compared to the second. For interpretation on the original scale, exponentiate the estimates. Signif. codes: <0.001 ‘***’ <0.01 ‘**’ <0.05 ‘*’

**Table 11:** Pairwise comparison of estimated marginal means obtained from GLM models of passage using only fibroblasts data.

Contrast	Ratio	SE	df	null	Lower CI (95%)	Upper CI (95%)	t-ratio	p-value	Significance
Early / Late	0.704	0.298	73	1	0.303	1.64	-0.831	0.4087	n.s

The row represents the contrast between two levels. 95% confidence interval (CI) and p-value are provided. P-value was adjusted for multiple comparisons using Tukey’s procedure. Signif. codes: <0.001 ‘***’ <0.01 ‘**’ <0.05 ‘*’

## 3 Results

**Figure 1:**
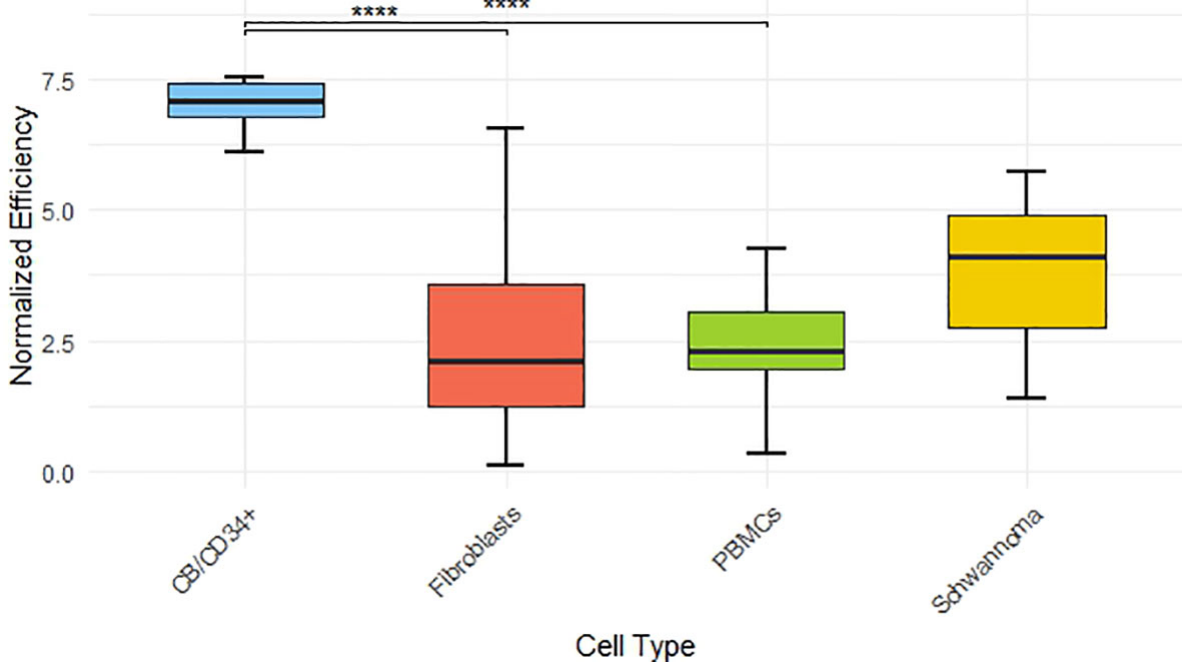
Normalized reprogramming efficiency across different cell types. Boxplots represent the distribution of normalized efficiency values for CB/CD34+, Fibroblasts, PBMCs, and Schwannoma cells. Significance codes: **** for p < 0.0001. *** for p < 0.001.** for p < 0.01. and * for p < 0.05.

**Figure 2:**
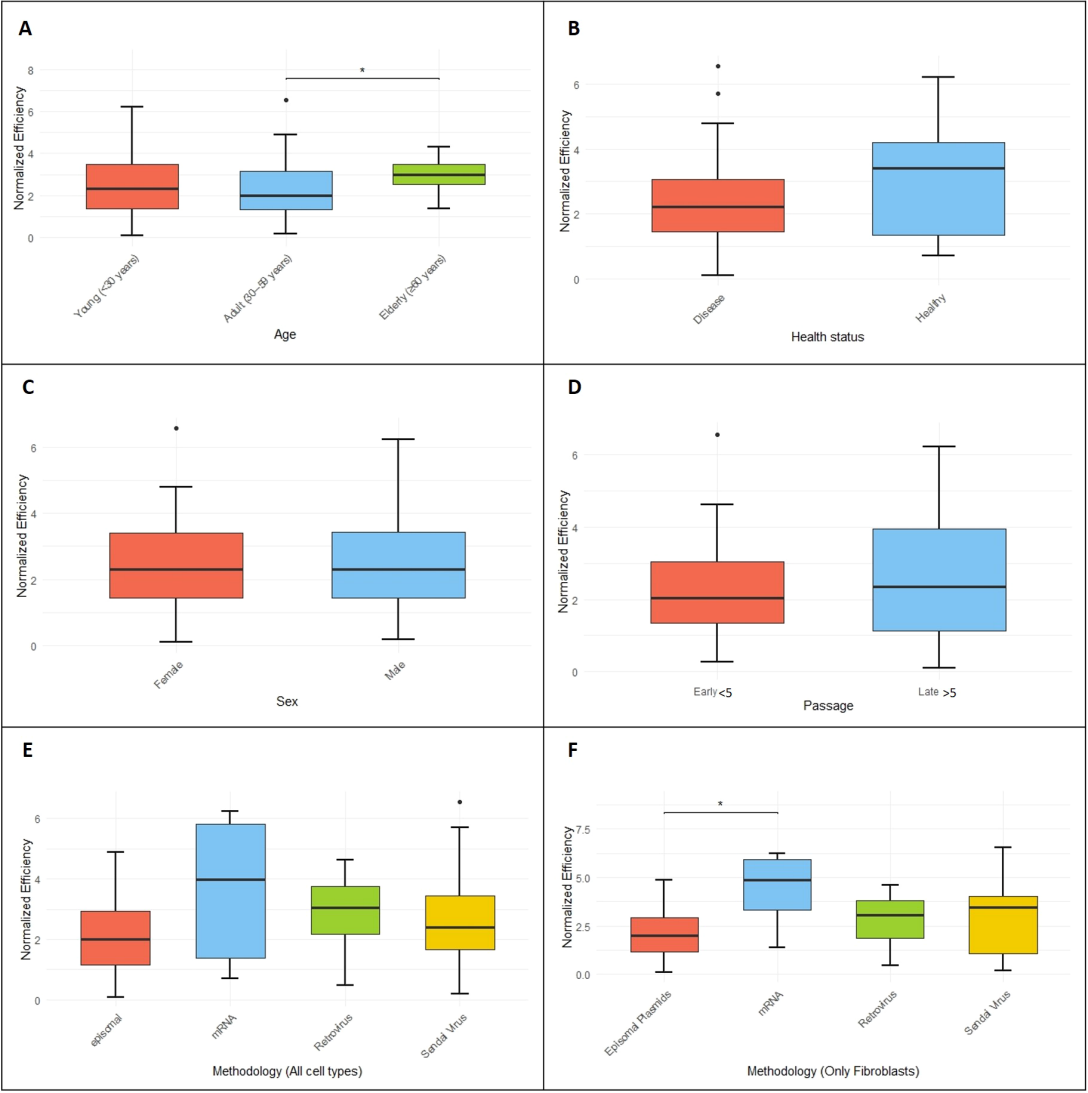
Normalized reprogramming efficiency across different biological and methodological variables. **(A)** Age group (Young, Adult, Elderly). **(B)** Health status (Healthy vs. Diseased). **(C)** Sex (Feminine vs. Masculine). **(D)** Passage number (Early vs. Late). **(E)** Reprogramming methodology applied across all cell types (Episomal plasmids, mRNA, Retrovirus, Sendai Virus). **(F)** Reprogramming methodology applied specifically to fibroblasts (Episomal plasmids, mRNA, Retrovirus, Sendai Virus). Boxplots represent the distribution of normalized efficiency values for each category within the analyzed variables. Significance codes: **** p < 0.0001, *** p < 0.001, ** p < 0.01, * p < 0.05.

To investigate whether specific parameters influence the reprogramming process, we calculated the mean ± standard deviation (SD) and median values to evaluate differences (**Table 2**). Additionally, we performed statistical analyses of reprogramming efficiencies across different donor and cell line characteristics to determine whether these differences were statistically significant (**Figures 1**, **2**).

Regarding the variable cell type, CD34^+^ cells isolated from cord blood exhibited the highest mean reprogramming efficiency (1.199% ± 0.529), compared to fibroblasts (0.040% ± 0.103), PBMCs (0.015% ± 0.013), and schwannoma cells (0.121% ± 0.158) (**Table 2**). This marked difference was confirmed by statistical analysis, with CD34^+^ cells showing the most significant differences (p < 0.0001) when compared to fibroblasts and PBMCs.

In normalized values, the efficiency of CD34^+^ cells was nearly five times greater than that of the other cell types. No significant differences were observed among the remaining cell types (**Figure 1**).

Among the other evaluated parameters, some statistically significant differences were observed (**Figure 2**). When comparing the three age groups, the mean reprogramming efficiencies were similar: young (0.039% ± 0.088), adult (0.027% ± 0.088), and elderly (0.024% ± 0.019). However, when comparing medians, young (0.009%), adult (0.006%), and elderly (0.019%), a higher reprogramming efficiency was observed in the elderly group (**Table 2**). This difference is consistent with the results of the Kruskal-Wallis test, which revealed a statistically significant difference (p < 0.05) between the elderly and adult groups, with the elderly group showing higher normalized reprogramming efficiency (**Figure 2A**). No significant differences were found between the other pairwise comparisons. In this analysis, CD34^+^ cell-derived lines were excluded from the young group to avoid bias due to their notably high reprogramming efficiency.

Focusing on the variable methodology, specifically when fibroblasts were used as the starting cell type, the mRNA-based technology showed a markedly higher mean reprogramming efficiency (0.221% ± 0.235), representing an order of magnitude increase compared to episomal plasmids (0.017% ± 0.027), retrovirus (0.030% ± 0.030), and Sendai virus (0.076% ± 0.170) (**Table 2**). This elevated mean is consistent with the statistical analysis, which showed that the mRNA-based method exhibited significantly higher reprogramming efficiency ($p$ < 0.05), particularly when compared to the episomal plasmid-based method (**Figure 2F**). In contrast, when methodologies were compared using the reprogramming efficiencies of all cell types, no statistically significant differences were found (**Figure 2E**), although the average reprogramming efficiency was lowest for episomal plasmids (0.017% ± 0.027; median = 0.006%) and highest for mRNA-based technology (0.177% ± 0.226; median = 0.052%).

Regarding the variables sex and passage number, both mean and median values showed minimal differences between groups. For sex, females had a mean of 0.029% ± 0.091 and a median of 0.009%, while males had a mean of 0.033% ± 0.075 and a median of 0.007% (**Table 2**). For passage number, early passage samples had a mean of 0.032% ± 0.110 and a median of 0.007%, compared to late passage samples with a mean of 0.049% ± 0.096 and a median of 0.010% (**Table 2**). In both cases, statistical analyses did not reveal any significant differences (**Figures 2C, D**, respectively).

For the health status variable, we found that the mean reprogramming efficiency for lines generated from healthy donors (Healthy) was 0.066% ± 0.116, with a median of 0.029%. In contrast, lines derived from patients (Disease) showed a mean of 0.024% ± 0.071 and a median of 0.008% (**Table 2**). Although no statistically significant differences were observed between these groups (**Figure 2B**), the mean reprogramming efficiency was slightly higher in healthy donors, and this difference was even more evident when comparing medians.

To explore potential confounding effects, complementary analyses were performed, using GLMs. Overall, these models yielded results consistent with those obtained using classical statistical approaches. No significant differences were detected among age groups (**Tables 3**, **5**, **8**), while samples from healthy donors exhibited higher reprogramming efficiencies compared to disease donors (**Tables 3**, **5**). Regarding methodology, analyses restricted to fibroblast-derived lines revealed higher efficiencies for Sendai Virus and mRNA compared to episomal plasmids (**Tables 9**, **10**). These findings support the robustness of our main conclusions and provide additional context without altering the overall interpretation of the study.

## 4 Discussion

In this study, the reprogramming efficiency of 150 iPSC lines generated at the BLC-B were compared and a statistical analysis was conducted, assessing the impact of various factors including the type of starting somatic cell, reprogramming method, passage number, donor age and sex, health status, and culture conditions.

Our findings indicate that CD34+ cells exhibit significantly higher reprogramming efficiencies (1.199% ± 0.529), compared to fibroblasts (0.040% ± 0.103), PBMCs (0.015% ± 0.013), and schwannoma cells (0.121% ± 0.158). This is likely attributable to their progenitor nature and inherent stemness, which facilitates the transition to pluripotency (46, 47).

At the BLC-B, fibroblasts have been the most commonly used cell type used for reprogramming, followed by PBMCs, which are increasingly employed as an alternative starting material for reprogramming. This trend is likely due to the less invasive nature of blood sample collection compared to skin biopsies. The optimal source of somatic cells for reprogramming into iPSCs depends on several factors, including ease of collection, reprogramming efficiency, and the intended use of the iPSCs (48, 49). Among these, fibroblasts are the most commonly used cell type due to the straightforward process of generating iPSCs from human skin (50). Hematopoietic sources, particularly CD34+ cells from bone marrow, cord blood, or peripheral blood, are believed to carry a lower risk of somatic mutations. These cells are minimally cultured or manipulated and have been reprogrammed using different techniques (50).

Concerning the reprogramming efficiencies observed across different methodologies, our results indicate that Sendai virus-based reprogramming using the CTS CytoTune™-iPS 2.1 Sendai Reprogramming Kit yielded the highest efficiency (1.199% ± 0.529). However, this outcome appears to be primarily influenced by the choice of cell type rather than the reprogramming method itself.

Upon comparison of reprogramming efficiencies across all cell types, excluding CD34^+^ cells, no statistically significant differences were observed among the methodologies. Nonetheless, evaluation of mean reprogramming efficiencies indicated that the mRNA-based approach yielded the highest efficiency, followed by the Sendai virus–mediated method, whereas the episomal plasmid–based strategy consistently exhibited the lowest efficiency (**Figures 2E, F**).

When focusing on reprogramming efficiencies using only fibroblasts as the starting cell type, the mRNA-based technology demonstrated a significantly higher mean efficiency, a result that was further supported by the normalized statistical analysis. This is in line with previously published studies (19, 51).

For example, Schlaeger et al. performed a comparative analysis of different non-integrating methods including mRNA-based reprogramming, SeV-based, and episomal systems, alongside lenti/retro viral generated iPSCs. All methods produced high-quality iPSCs but exhibited significant differences in reprogramming efficiency. Among the non-integrating approaches, mRNA-based reprogramming showed the highest efficiency, followed by SeV based methods (51).

Another comparative study by Manzini et al., using 3 different patient derived fibroblast lines reprogrammed with SeV or episomal plasmids, found that SeV based reprogramming was overall the most efficient method. However, the reprogramming efficiency was intrinsically dependent on the individual fibroblast lines, highlighting the cell line-dependent variability in reprogramming potential (52).

The Biobanking department of the Coriell Institute (Camden, New Jersey) recently published a study, comparing reprogramming success rates between nucleofection with episomal plasmids and SeV transduction. They reviewed a total of 50 cell lines reprogrammed with the SeV method and 26 cell lines reprogrammed with the episomal method using several different starting cell types like fibroblasts, lymphoblastoid cells lines (LCLs), and PBMCs. In their hands, SeV reprogramming demonstrated a significantly higher reprogramming success rate compared to the episomal reprogramming method regardless of the source material (53).

Furthermore, there are several studies published that compare reprogramming methodologies in general (36, 54–58) or more specific in terms of safety, efficiencies and potential for clinical application (59, 60).

In summary, using non-integrating Sendai virus to transduce somatic cells appears to be the best approach for producing iPSCs. Sendai virus strikes a balance between high efficiency, safety, and clinical applicability, which makes it a preferred method for generating iPSCs in a clinical setting (59). Its non-integrating nature, transient expression, and ability to avoid genomic alterations address major concerns associated with other reprogramming techniques, particularly in the context of long-term safety and tumorigenesis. Sendai Virus genome or transcripts can easily be removed from the cells due to a temperature sensitive mutation. The temperature-sensitive mutation in the Sendai virus backbone provides greater control over the duration of reprogramming factor expression.

GMP-grade Sendai virus reprogramming kits are available where c-Myc is substituted by L-Myc ensuring a safer profile for potential therapeutic applications and are being used to produce clinical-grade iPSCs (39, 61). RNA reprogramming is generally more efficient; a key limitation is that it typically requires cells to be adherent for effective transfection and can therefore not be used for reprogramming of CD34+ cells or PBMCs (61).

Although reprogramming efficiency is generally considered to decline with chronological age, our data indicates higher efficiencies in elderly donors compared with adults-despite the elderly group containing only 20 samples-and a trend toward increased efficiency in younger donors. This suggests that age-associated effects on cellular plasticity are more nuanced than previously appreciated. Prior studies have reported reduced reprogramming in aged somatic cells (62–66), but other reports highlight substantial donor-to-donor variability and indicate that biological age, rather than chronological age, may better predict reprogramming capacity (67, 68). This apparent discrepancy may reflect heterogeneity in biological aging, as immune and inflammatory states—both of which strongly influence reprogramming—can vary widely within age groups (38, 69). Residual technical or donor-related factors may also contribute despite our attempts to control for them. Notably, emerging evidence shows that aging-associated epigenetic changes or stress responses may, in certain contexts, facilitate reprogramming, providing a plausible biological basis for our observation (69, 70). These findings highlight the need for deeper integration of immunological, epigenetic, and donor-specific variables to fully understand age-related differences in iPSC generation.

Another noteworthy observation is the tendency for higher reprogramming efficiencies in cell lines derived from healthy donors (0,066%) compared to those from patients (0,024%). This difference may be attributed to disease-related alterations in cellular pathways, which vary among individuals and could impair the reprogramming process.

A limitation of this study is the imbalance in the number of cell lines across groups. This uneven sampling reduces statistical power for certain comparisons and limits the ability to fully assess variability within smaller groups.

Another limitation of our analysis is the potential association between cell type and reprogramming method, which could introduce confounding effects. To minimize this, CD34+ samples were excluded, as these cells were exclusively reprogrammed using the CTS CytoTune™-iPS 2.1 Sendai Reprogramming Kit and exhibit markedly higher efficiencies. After this exclusion, the dataset included fibroblasts and PBMCs, with fibroblasts reprogrammed using multiple methods, allowing a fair comparison of methodologies. PBMCs were primarily associated with the CytoTune™-iPS 2.0 Sendai Reprogramming Kit, which we acknowledge as a residual source of linkage; therefore, separate analyses for methodology (all cell types vs. fibroblasts only) were performed, For donor-related variables such as age and sex, the full dataset was used because these characteristics are independent of cell type and methodology and reflect intrinsic donor factors. While multivariate models (e.g., GLMs or mixed-effects models) could further account for interactions, the sample size and exploratory nature of this study led us to prioritize classical approaches for clarity and reproducibility. Future studies with larger and more balanced datasets should incorporate multivariate modeling to fully disentangle these relationships.

Furthermore, a complementary analysis using GLMs was conducted, providing a framework to model the joint influence of multiple variables and the underlying distribution of the data. Overall, the GLM results were broadly consistent with those obtained using classical statistical approaches, supporting our findings. Minor differences were observed, likely reflecting the way GLMs handle multivariate structure. Specifically, GLM estimates indicated no differences among age groups (**Tables 3**, **5**, **8**), whereas samples from healthy donors exhibited higher reprogramming efficiencies compared to those from disease donors (**Tables 3**, **5**). Another interesting finding concerns the significant difference between some methodologies. This was only evaluated using fibroblast-derived data, as this cell type was reprogrammed by all methodologies. Our results indicate that mRNA and Sendai Virus generate iPSCs with higher efficiency than episomal plasmids (**Tables 9**, **10**).

Among the numerous factors influencing reprogramming efficiency, the intrinsic developmental state of the starting somatic cells stands out as one of the most critical determinants. Cells that retain a more plasticity or progenitor-like state tend to reprogram more readily compared to fully differentiated cells, likely due to their more accessible chromatin landscape and active expression of genes associated with self-renewal. While external variables such as culture conditions, reprogramming methods, and passage number can influence outcomes to some extent, they often play a secondary role in comparison to intrinsic cellular characteristics. Inherent donor-specific biological characteristics have emerged as a major factor shaping reprogramming efficiency, with certain genetic variants potentially affecting key pathways related to cell cycle regulation, epigenetic remodeling, or response to reprogramming factors (71–73).

## Data Availability

The raw data supporting the conclusions of this article will be made available by the authors, without undue reservation.
