# Supplementary material for: Deciphering reprogramming efficiency in human induced pluripotent stem cells: insights from the generation of 150 cell lines
Source: Front Immunol. 2026 Jan 13;16:1719056. doi: 10.3389/fimmu.2025.1719056 (PMC12834790; doi:10.3389/fimmu.2025.1719056)

**Supplementary Figure 1.** Exploratory analysis of the efficiency variable to guide GLM specification. A) Histogram of efficiency values. B) Relationship between variance and mean efficiency.


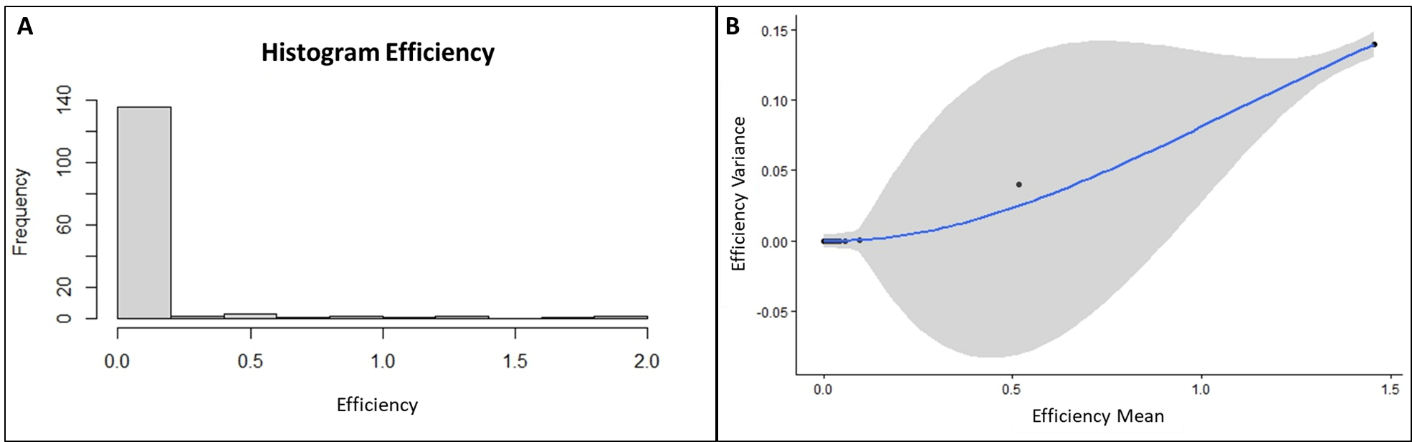


**Supplementary Figure 2.** Diagnostic plots for the selection of family and link in the generalized linear model (GLM) using all data. A) Residual Q-Q plot. B) Residuals vs fitted values. Results from DHARMa residual tests in R: KS test p = 0.98 (no significant); dispersion test p < 0.05 (significant deviation); outlier test p = 1 (no significant).


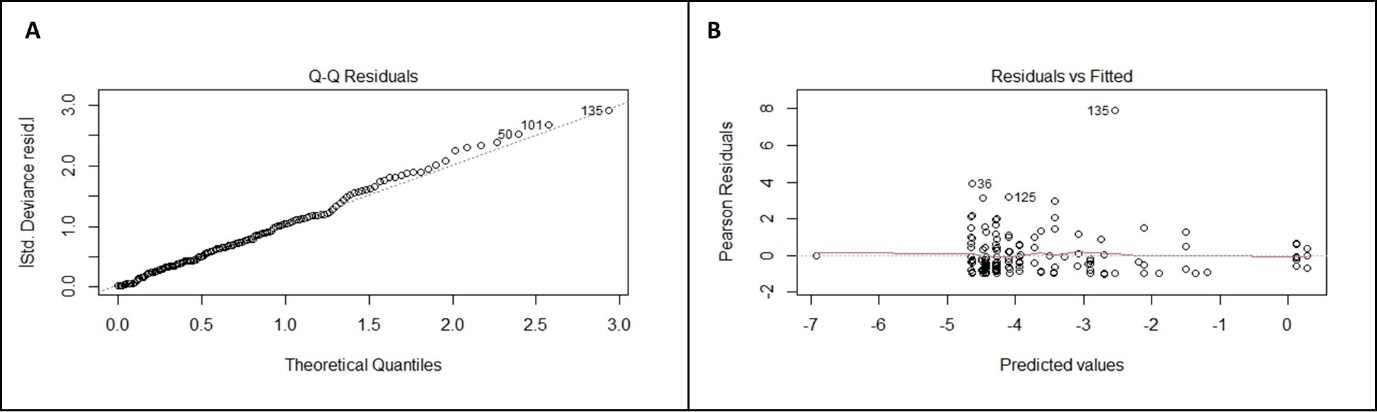

Supplement: Supplementary file 1 [file DataSheet1.docx]
